# Supplementary material for: Cranial morphology of Sinovenator changii (Theropoda: Troodontidae) on the new material from the Yixian Formation of western Liaoning, China
Source: PeerJ. 2018 Jun 20;6:e4977. doi: 10.7717/peerj.4977 (PMC6015489; doi:10.7717/peerj.4977)
Supplement: Supplemental Information 1 [file peerj-06-4977-s001.docx]

**APPENDIX**

**Updated Information for Phylogenetic Analysis**

The data matrix is modified from Xu et al.’s (*2015*) analysis. Two states are added to Character 6 and Character 8 as following:

Character 6: Otosphenoidal crest vertical on basisphenoid and prootic, and does not border an enlarged pneumatic recess (0) or present, crescent shaped crest forms the anterodorsal edge of an enlarged pneumatic recess, and ventral to CN VII (1) or well developed, crescent shaped, thin crest forms anterior edge of enlarged pneumatic recess, and dorsal to CN VII (2).

PMOL-AD00102, *Sinovenator*, and *Byronosaurus* are coded as 1 for this character. All other troodontids previously coded as "1" for this character are recoded as "2".

Character 8. Subotic recess (pneumatic fossa ventral to fenestra ovalis) absent (0) or present as a shallow concave (1) or present as a deep socket (2).

PMOL-AD00102 and *Sinovenator* are coded as "1" for this character. All other troodontids previously coded as "1" for this character are recoded as "2".

PMOL-AD00102 as a separate taxon.

?0??010100000011120?????10???0?00110020120000210?1011011110111101??01???00111100??01?100????0111?1?1???????????????????????????????????????????????????????????????????????????????????????????????????????????10???1???0?0?110????0???????1????001?????1011??10??????????????????????????????????????????????????????????????????????????????????????0???1?????00000?0?????010????1??

PMOL-AD00102 provides new information for *Sinovenator*, and a total 52 characters of *Sinovenator* are updated.

?0??0101[02]000001112001010101110000110020120000[12]10?101101[01]100111101000100100111100000111[01]01???0111?1110001101000010001102???1?????????010111?0??????????000?0?21??0111002000211011100?11110000???0010001011??000?10??011??00001100??00?301100110?0001?010?101100100?000?1111??00100???????????011?0????0000000???2011011?101?1???0011??0??1000112?00??000??110??1100000?0000100100000111

*Sinusonasus*

?0?????????????????0101???11100??????201???????????????????????????0?001????????0?01110?1????????????????????0????01?02211??????????????????????????????????2???????002?0???1?0????????1???0?????1?00??21?0?0???????????0??????0???0?301100?101000??0?00????1?0???0?0?????1?????????????????????????????????????0110110101?100000110????10???021?0??0?0?????1???????????00100??0??0111

**REFERENCES**

Xu X, Zheng X-T, Sullivan C, Wang X-L, Xing L-D, Wang Y, Zhang X-M, O’Connor JK, Zhang F-C, Pan Y-H. 2015. A bizarre Jurassic maniraptoran theropod with preserved evidence of membranous wings. *Nature* **521:**70–73DOI 10.1038/nature14423.
